# Differentiation of malignant and benign lung lesions with diffusion-weighted MR imaging

**DOI:** 10.2478/v10019-012-0021-3

**Published:** 2012-04-11

**Authors:** Sevtap Gümüştaş, Nagihan Inan, Gür Akansel, Ercüment Çiftçi, Ali Demirci, Sevgiye Kaçar Özkara

**Affiliations:** 1 Department of Radiology; 2 Department of Pathology, School of Medicine, University of Kocaeli, Turkey

**Keywords:** pulmonary lesions, diffusion-weighted imaging, apparent diffusion coefficient, magnetic resonance imaging

## Abstract

**Background:**

The aim of the study was to evaluate the role of diffusion-weighted magnetic resonance imaging in the differential diagnosis of lung lesions.

**Patients and methods.:**

Sixty-seven patients with lung lesions (48 malignant, 19 benign) were included in this prospective study. Signal intensities (SIs) were measured in diffusion-weighted MR images that were obtained with b=0, 500 and 1000 s/mm^2^ values. Apparent diffusion coefficient (ADC) maps were calculated by using images with b=0 and 1000 s/mm^2^ values. The statistical significance was determined using the Student-t test.

**Results:**

The SIs of malignant lesions were significantly higher than those of benign lesions (p<0.004 for b=0 s/mm^2^ and p<0.000 for the other b values). Using b=500 s/mm^2^, SI≥391 indicated a malignant lesion with a sensitivity of 95%, specificity of 73% and positive predictive value of 87%. Using b=1000 s/mm^2^, SI≥277 indicated a malignant lesion with a sensitivity of 93%, specificity of 69% and positive predictive value of 85%. There was no significant difference between malignant and benign lesions regarding ADC values (p=0.675). There was no significant difference in SIs or ADC values between small cell carcinoma and non-small cell carcinoma. When comparing undifferentiated with well- partially differentiated cancers, SIs were higher with all b values, but the difference was statistically significant only with b=1000 s/mm^2^ (p<0.04).

**Conclusions:**

Diffusion-weighteted MR trace image SI is useful for the differentiation of malignant versus benign lung lesions.

## Introduction

Lung cancer is one of the leading causes of death.^1^ It usually arises as a solid nodule or mass on chest radiography or computed tomography (CT). Although many well known characteristics have been described for nodule differentiation on CT, it remains a challenge for radiologists to differentiate lesions as malignant or benign.^2^–^5^ In recent years, fluorine-18 fluorodeoxyglucose positron emission tomography (PET) has been used for this purpose. Both CT and PET deliver high doses of radiation. In addition PET has been known to give false-positive results in inflammatory masses.^6^–^9^ For these reasons, an accurate and safe alternative method is still desirable for the determination of malignant versus benign pulmonary lesions.

Recent advances in fast imaging techniques like echo-planar imaging, makes magnetic resonance imaging (MRI) more suitable for chest applications.^10^–^12^ There are reports using dynamic contrast MRI of lung masses.^13^,^14^ Diffusion-weighted magnetic resonance imaging (DWI), initially used in the central nervous system, has been increasingly applied in other body areas, such as the mediastinum^15^, pancreas^16^, and liver.^17^,^18^

The aim of our study was to determine whether quantitative analysis of DWI could be helpful in the differentiation of malignant and benign pulmonary lesions.

## Patients and methods

### Patients

The study was approved by our institutional review board and protocol review committee. Because the tests used were a part of the routine clinical workup of these patients, the informed consent was not required by the review board. We obtained a blanket consent from all patients for the use of their findings for research and educational purposes, with patient privacy secured.

From March 2009 to July 2010, 66 consecutive patients (49 males, 17 females) with 67 lung lesions found on CT were included in this prospective study according to our entry criteria. The entry criteria were: (a) presence of a solid pulmonary lesion. In the case of ground glass opacity (GGO) with the solid part on CT images, the GGO part was avoided to the extent possible and the solid part was measured on DWI with the reference of CT images, since air itself in GGO might reduce the true apparent diffusion coefficient (ADC) value of the lesion.^18^ Lesions containing large amounts of necrosis and calcification were also excluded; (b) lesion size >10 mm in diameter in view of the limited planar resolution of DWI; (c) presence of a specific proven diagnosis of the lesion either histopathologically or by using clinical, radiological and laboratory data or based on at least 1-year radiological follow-up; (d) no current administration of chemotherapeutic or radiotherapeutic treatment; (e) absence of any contraindications for MRI; and (f) ability of patients to lie still and hold their breath approximately 26 seconds in the MRI unit.

Biopsies of lung lesions were carried out by the radiologist (EÇ) in the interventional radiology department of the same hospital. MRI was performed on these patients on the same day but before the percutaneous biopsy in order to avoid hemorrhage-related distortions.

The study was carried out according to the Helsinki Declaration.

### MR Imaging

All patients were examined with a 1.5 Tesla MR unit (Gyroscan Intera; Philips Medical Systems, Eindhoven, The Netherlands) using a four-element phased-array body coil. This system had a maximal gradient strength of 30 mT/m and a slew rate of 150 mT/m/msec. All patients were examined initially with the routine MRI protocol for the thorax that included: precontrast axial T1-weighted (W) breath-hold spoiled gradient echo (fast field echo: FFE) (TR/TE/FA/NEX:169/4.6/80/1) and coronal and axial T2-W single-shot turbo spin-echo (TR/TE/NEX/TSE factor: 700/80/1/72). Subsequently, three series of axial single-shot spin-echo echo-planar (SS-SE-EPI) DWI (1,000/81; echo-planar imaging (EPI) factor, 77; sensitizing gradients in *x*, *y*, and *z* directions) were acquired using b = 0, 500 and 1000 s/mm^2^ values. ADC maps were reconstructed from the b = 0 and b = 1000 s/mm^2^ images. MRI, including DWI, consisted of a multisection acquisition with a slice thickness of 6 mm, an intersection gap of 1 mm, and an acquisition matrix of 128 × 256. All sequences were acquired using a partially parallel imaging acquisition and SENSE (sensitivity encoding) reconstruction with a reduction factor (*R*) of 2. The scanning time of the acquisition of each DWI series during a single breath-hold was 26 seconds.

### Quantitative analysis

Quantitative measurements were made using a dedicated Workstation (Dell Workstation Precision 650, with the View Forum software platform provided by Philips Healthcare). All images were assessed by two radiologists (SG, NI) who were blinded to the clinical history of the patients. First, CT images were evaluated in order to assess the calcification, necrosis and GGO components. CT scans were also evaluated for contour characteristics of the lesions (irregular or smooth) and concomitant interstitial findings were recorded. Those findings were compared with the DWI findings. The range of interval between the CT and MRI examinations was 0–10 days (mean, 5. 6 days). Then, the lesion was visualized once more on the conventional T1-W and T2-W MRI in terms of location, size and content of cystic-necrotic parts to detect interval changes since the time of the CT scan. These conventional images were only used for the lesion identification and not for the analysis. Afterwards, signal intensity (SI) of the lesion was measured for each b value (0, 500 and 1000 s/mm^2^) on DWI using a round or elliptical region of interest (ROI). The ROI was placed centrally, and the size of the ROI was kept as large as possible, covering at least two-thirds of the lesion, yet avoiding the interference from the surrounding lung tissue, necrotic parts and major blood vessels. ADCs were then calculated from the ADC maps that were reconstructed from b = 0 and b = 1000 s/mm^2^ values.

### Statistical analysis

SIs and ADCs were compared between the groups. The fitness of numeric data set to normal distribution was determined using the Kolmogorov-Smirnov test. The data were normally distributed, so the differences in SIs and ADCs were analyzed using the Student-t test. A *p* value of less than 0.05 was considered statistically significant. To evaluate the diagnostic performance of the quantitative tests for differentiating malignant and benign lesions and to describe the sensitivity and specificity of the tests, receiver operating characteristic (ROC) analysis was performed. The optimum cutoff value was determined as the value that best discriminates between the two groups in terms of maximum sensitivity and minimum number of false-positive results. All statistical analyses were performed using the statistical software SPSS.^15^

## Results

The mean age of all patients was 64 ± 21 years (range: 41–83 years). The mean age of the patients in the benign group was 61 ± 19 years (range: 41–72 years) and in the malignant group was 66 ± 21 years (range: 44–83 years). The difference in age between groups was not statistically significant (*p* = 0.5).

The mean diameter of masses for the entire group was 33 ± 18 mm. The mean diameter of malignant lesions was 37 ± 19 mm (range: 20–130 mm) and of benign lesions was 25 ± 15 mm (range: 15–60 mm), respectively. The difference in diameter between groups was statistically significant (*p* = 0.005). Except for one patient who had two nodules (the primary tumor and its pulmonary metastasis), one lesion was measured in the remaining patients.

Of the 67 lesions, 48 were malignant and 19 were benign (Table 1). The malignant lesions consisted of 42 non-small cell lung cancers (NSCLCs) and 6 small cell lung cancers (SCLCs). NSCLC subgroups included 13 adenocarcinomas, 5 squamous-cell carcinomas, 2 large-cell carcinomas, and 22 unidentified NSCLCs that could not be subgrouped in any of these. Eleven of the malignant lesions were poorly-differentiated, the others were medium- or well- differentiated. The benign lesions consisted of 9 chronic inflammatory changes, 3 sarcoidosis, 2 acute bacterial pneumonias, 2 tuberculosis pneumonias, 1 rheumatoid nodule, 1 granuloma, and 1 hamartoma.

In 51 patients, the final diagnosis was made by histopathological confirmation on the basis of percutaneous biopsy, and included 48 primary lung cancers, 2 nonspecific inflammatory changes and 1 hamartoma. The diagnosis was confirmed with laboratory, radiological and clinical parameters in 3 sarcoidosis, 2 bacterial pneumonias, and 2 tuberculosis pneumonias. In sarcoidosis patients, the lesions regressed with a specific treatment. Two tuberculosis (Mycobacterium tuberculosis) and 2 bacterial pneumonia cases were diagnosed bacteriologically. In 9 patients (1 granuloma, 1 rheumatoid nodule and 7 chronic inflammatory changes) lesions remained stable on follow-up CT for 12 months or more.

**FIGURE 1:**
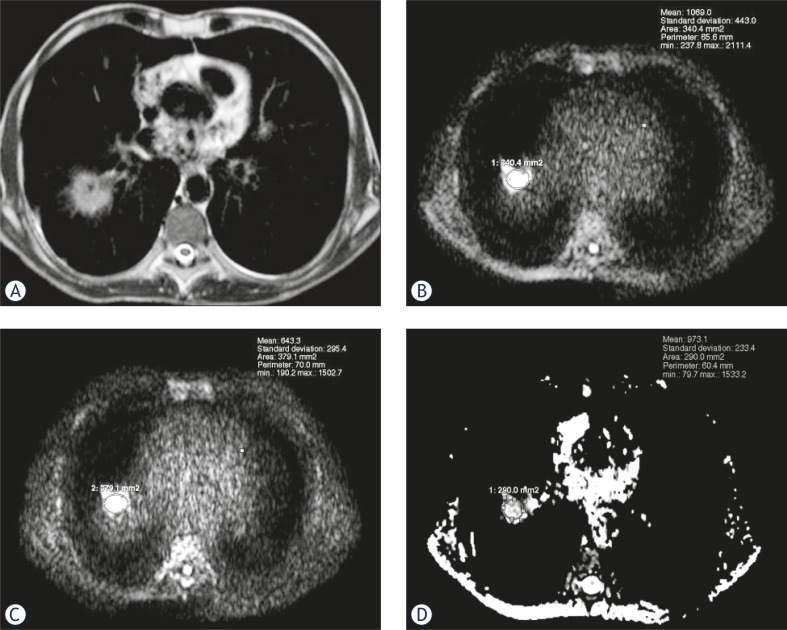
A 56-year-old man with poorly-differentiated non-small cell carcinoma. (A) Axial T2-weighted MR image shows mass in the right lung. (B) diffusion-weighted magnetic resonance image with b value of 500 sec/mm^2^ shows hyperintense mass (SI = 1069). (C) diffusion-weighted magnetic resonance image with b value of 1000 sec/mm^2^ shows hyperintensity of the mass is still remarkable (SI = 643). (D) ADC map shows ADC value is (0.97×10^−3^) sec/mm^2^.

**FIGURE 2:**
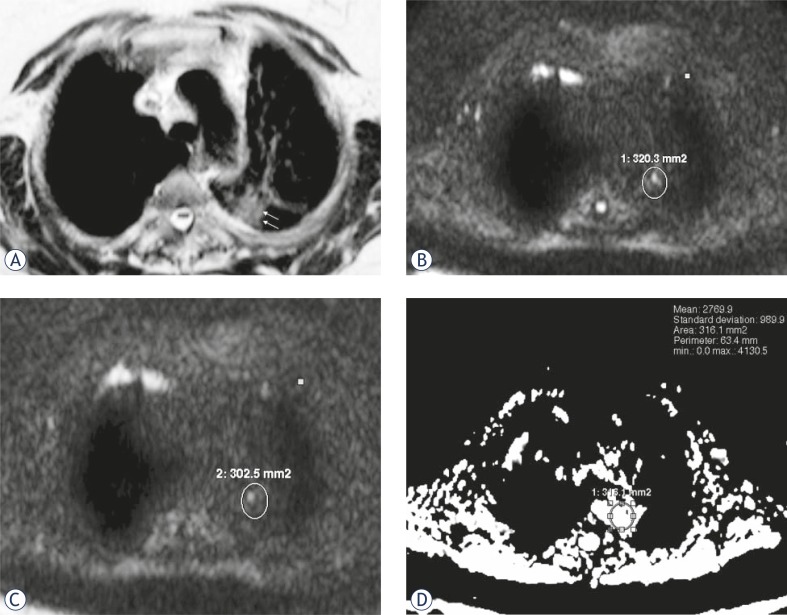
A 32-year-old woman with bacterial pneumonia. (A) Axial T2-weighted MR image shows ill-defined peripheral mass in the superior segment of the lower lobe of the left lung (arrows). (B) diffusion-weighted magnetic resonance image with b value of 500 sec/mm^2^ shows minimal hyperintense mass (SI = 203). (C) diffusion-weighted magnetic resonance image with b value of 1000 sec/mm^2^ shows hyperintensity of the mass is less remarkable (SI = 162). (D) ADC map shows ADC value is (2.76×10^−3^) sec/mm^2^.

**FIGURE 3:**
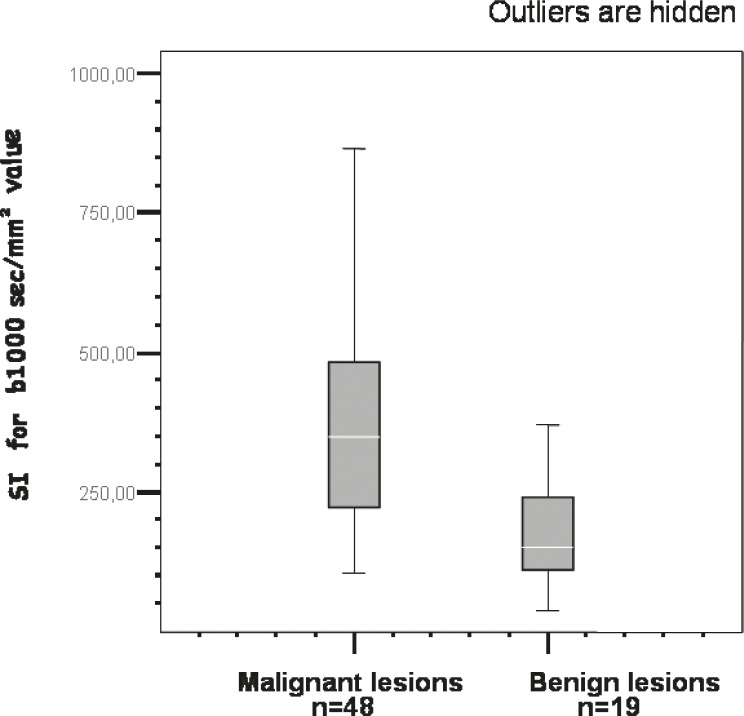
Comparison of SI with b value of 1000 s/mm^2^ in malignant and benign lesions. The SIs of malignant lesions were significantly higher than those of benign lesions.

We could obtain DWI SI and an ADC value for all lesions. The results of the quantitative analysis of SIs and ADC values are reviewed in Table 2. The mean SI of malignant lesions was higher than that of benign lesions (Figure 1, 2). The difference between malignant and benign lesions was significant for all b values (*p* < 0.004 for b = 0 s/mm^2^ and *p* < 0.000 for the other b values) (Figure 3). The area under the ROC curve was 0.933 ± 0.031 for the SI on images with b = 500 s/mm^2^. Using b = 500 s/mm^2^, a SI ≥391 indicated a ma lignant lesion with a sensitivity of 95%, specificity of 73% and positive predictive value of 87%. Six among 48 malignant lesions (4 NSCLCs and 2 SCLCs) revealed SIs <391, which could be confused as benign.

For the SI on images with b = 1000 s/mm^2^, the area under the ROC curve was 0.831 ± 0.055. A SI ≥227 with b = 1000 s/mm^2^ indicated a malignant lesion with a sensitivity of 93%, specificity of 69% and positive predictive value of 85%. Seven among 48 malignant lesions (6 NSCLCs and 1 SCLCs) revealed SIs <227 which could be confused as benign. Among the benign lesions, 1 sarcoidosis and 1 acute bacterial pneumonia had SI higher than the cut-off value on DWI with both b = 500 and b = 1000 s/mm^2^. A patient with a chronic inflammatory lesion had a SI higher than the cut-off value only for b = 1000 s/mm^2^. Although the mean ADC of the malignant lesions (1.5 ×10^−3^ mm^2^/sec) was lower than of the benign group (1.9 ×10^−3^ mm^2^/sec), the difference was not statistically significant (*p* < 0.675).

The results of the subgroup quantitative analysis are reviewed in Table 3. When we analyzed the malignant lesions in accordance with the histologic differentiation, the SI of poorly-differentiated cancers was higher for all b values, but statistically a significant difference was observed only with b = 1000 s/mm^2^ (*p* < 0.04). Although the ADC of poorly-differentiated lesions was lower than of the medium-well differentiated lesions, the difference was not statistically significant (*p* < 0.240). A comparison of the NSCLCs and the SCLCs demonstrated that the SIs of SCLCs were higher than those of NSCLCs. Although the ADC value of the SCLCs was lower than the ADC value of the NSCLCs, the difference was not statistically significant (*p* < 0.464) (Table 4).

When we compare CT images with the DWI, all of the malignant lesions had irregular contours on CT images. Of the benign lesions 16 had also irregular contours but 3 (1 rheumatoid nodule, 1 granuloma, and 1 hamartoma) had smooth contours. The difference was not statistically significant (p = 0.3). Lymphangitic tumoral spread as irregular septal thickening was detected as a concomitant interstitial finding in 6 of the malignant masses on CT images.

## Discussion

The aim of DWI is to evaluate the diffusion process *in vivo*. ADC values are quantitative expressions of the diffusion characteristics of tissues. These characteristics are related to several factors such as tissue cellularity, cell density and extracellular-intra-cellular components.^16^ DWI has been an important diagnostic tool for neuroradiology, especially for ischemic events of the brain.^19^ Although DWI has been used to differentiate malignant and benign lesions in several other locations, there are few studies about the intrathoracic lesion characterization.^20^,^21^–^27^

In our study, we found SIs of malignant masses on diffusion trace imaging were significantly higher than of benign masses with low (b = 500 s/mm^2^) and high (b = 1000 s/mm^2^) b values. In the study of Uto *et al.* the SI ratio of malignant lesions to the spinal cord was found higher than that of benign lesions.^21^ Satoh *et al.* also found a higher qualitative score in SI of malignant versus benign masses but they did not measure quantitatively.^22^ In our study, the patient population was larger than in these two studies.

Although malignant lesions showed lower ADC values than benign masses in our study, the difference was not significant. One reason may be the use of fixed TRs, due to which the DW series were acquired in different phases of the cardiac cycle. Therefore, DW images of different patients may have been affected differently by the pulsatile motion. Another reason may be the distortion artifacts, which limited the reliability of the ADC measurements for small and low-lateral segmental lesions. In the literature, some studies did not evaluate ADC values probably because of susceptibility artifacts.^22^,^25^ Uto *et al.* found no significant difference between malignant and benign lesions by means of ADC.^21^ Mori *et al.* found a significant difference between malignant and benign lesions by using an ADC cut-off value of 1.1×10^−3^ mm^2^/sec.^23^ The large number of patients may be the explanation for this result. Liu *et al.* reported that ADC values in malignant lesions were significantly lower than in benign lesions.^24^ In the same study, there was no significant difference between malignant and benign lesions in DWI SIs.

Histopathologically, tumoral cellularity of SCLC is high, and these tumoral cells have very large nuclei and almost no cytoplasm.^28^ All these features were expected to restrict the tissue diffusion and reduce ADC values. In our study, the SI of SCLC lesions was higher than of the NSCLC subgroup but the difference was significant only with b = 0 s/mm^2^. Thus, hyperintensity of lesions may be related to the T2 shine-through effect. Although ADCs were lower in the SCLC subgroup, the difference was not statistically significant. This may be because of the limited number of patients (n=6). In their study of tissue characterization in lung cancers, Abdel Razek *et al.* found significantly lower ADC values for SCLC when comparing with NSCLC groups in a similar patient population.^26^ Liu *et al.* also found lower ADC values for the SCLC than the NSCLC group.^24^ However, Koyama *et al.* found no significant differences between sub-types of lung cancers.^27^

In our study, SI of poorly- differentiated malignant masses was higher than of medium-well differentiated masses on all trace images and the difference with b value of 1,000 s/mm^2^ was statistically significant. ADC values were lower in the poorly-differentiated subgroup although the difference was not statistically significant. Histologically, tumor cellularity is higher in the poorly-differentiated cancers which could explain the low ADC values.^29^ Similar results of significantly lower ADCs in poorly-differentiated adenocarcinomas compared medium-well- differentiated cancer types are reported in the literature and are in accordance with our findings.^20^,^26^,^30^,^31^

When compared with CT images, contour characteristics of the lesions cannot be assessed with DWI but it does not seem to be a significant disability because contour characteristics are not reliable in differentiating malignant and benign lesions. On CT images lymphangitic tumoral spread was detected as a concomitant interstitial finding in some of the malignant masses. This finding is very helpful to estimate the malignant character of the lesion. The sensitivity and specificity achieved by DWI suggest it could be used for malignant versus benign differentiation. However, the inability of MRI to properly assess the interstitium and lymphangitic tumoral spread is a limitation for predicting malignancy. In addition, although just primary lung cancers are involved into our study, it must be kept in mind that calcified metastases such as those from osteosarcoma may be difficult to detect because of relatively lower proton density resulting in low signal intensity.

Our study had some technical limitations. The use of DWI in the thorax was hindered by certain limitations such as physiologic motion artifacts (respiration and cardiac motion), low signal-to-noise ratio (SNR) of the low lung proton density and the susceptibility artifacts caused by air-tissue interfaces.^18^ We used the EPI sequence with high b values, which had a lower SNR, thus resulting in greater image distortion. In addition, we obtained DW images using a breath-hold echo-planar sequence with SENSE and this made the measurements vulnerable to susceptibility effects. We did not use pulse-triggered DWI, known to reduce the accuracy of ADC measurements.^18^ Finally, the patient population, especially the benign subgroup, was relatively small, which might compromise the accuracy of the results.

In conclusion, DWI may be used to differentiate malignant and benign lung lesions in addition to other radiologic imaging techniques even with high b values such as b = 1000 s/mm^2^. DW trace image SI, together with ADC measurements is useful for the differentiation of malignant versus benign lung lesions.

## Figures and Tables

**TABLE 1:** Histopathological and clinical diagnosis of lesions

	**Diagnosis**
Benign lesions (n=19)	Chronic inflammatory changes (n=9)
	Sarcoidosis (n=3)
	Acute bacteriel pneumonia (n=2)
	Tuberculosis pneumonia (n=2)
	Romatoid nodule (n=1)
	Granuloma (n=1)
	Hamartoma (n=1)
Malignant lesions (n= 48)	SCLC (n=6)
	NSCLC (n=42)
	NSCLC subgroup;
	Adenocarcinoma (n=13)
	Squamous cell carcinoma (n=5)
	Large cell carcinoma (n=2)
	Nonidentified NSCLC (n=22)

SCLC = small cell lung cancer, NSCLC = non-small cell lung cancer

**TABLE 2:** Quantitative analysis of diffusion-weighted magnetic resonance imaging (mean ± SD) of malignant and benign lesions (b=0, 500, 1000 sec/mm^2^ and ADC values)

**b and ADC values**	**malignant lesions (n=48)**	**benign lesions (n=19)**	***P***
b 0 (sec/ mm^2^)	1500.40 ± 820	960.31 ± 550	*0.004*
b 500 (sec/ mm^2^)	611.32 ± 343	247.10 ± 124	*0.000*
b 1.000 (sec/ mm^2^)	371.00 ± 194	175.68 ± 94	*0.000*
ADC (×10^−3^) mm^2^/sec	1.65 ± 0.90	1.75± 0.70	0.675

**TABLE 3:** Quantitative analysis of diffusion-weighted magnetic resonance imaging (mean ± SD) of poorly-differentiated and medium- well differentiated malignant lessions (b=0, 500, 1000 sec/mm^2^ and ADC values)

**b and ADC values**	**poorly-differentiated (n=11)**	**medium-well differentiated (n=31)**	***P***
b 0 (sec/ mm^2^)	1646 ± 787	1453.3 ± 837	0.497
b 500 (sec/ mm^2^)	751.4 ± 440	568.87 ± 304	0.245
b 1.000 (sec/ mm^2^)	517.27 ± 273	323.67 ± 135	*0.044*
ADC (×10^−3^) mm^2^/sec	1.5 ± 0.5	1.8 ± 0.8	0.240

**TABLE 4:** Quantitative analysis of diffusion-weighted magnetic resonance imaging (mean ± SD) of non-small cell lung cancer (NSCLC) and small cell lung cancer (SCLC) (b=0, 500, 1000 sec/mm^2^ and ADC values)

**b and ADC values**	**NSCLCs (n=42)**	**SCLCs (n=6)**	***P***
b 0 (sec/ mm^2^)	1110.8 ± 209	1705 ± 873	*0.001*
b 500 (sec/ mm^2^)	468.6 ± 238	646.9 ± 350	0.186
b 1.000 (sec/ mm^2^)	366 ± 177	379.1 ± 197	0.881
ADC (×10^−3^) mm^2^/sec	1.9 ± 0.8	1.5 ± 1.0	0.464

## References

[1] Jemal A, Siegel R, Ward E, Murray T, Xu J, Smigal C (2006). Cancer statistics. CA Cancer J Clin.

[2] Erasmus JJ, Connolly JE, McAdams HP, Roggli VL (2000). Solitary pulmonary nodules. Part 1. Morphologic evaluation for differentiation of benign and malignant lesions. RadioGraphics.

[3] Swensen SJ, Viggiano RW, Midthun DE, Müller NL, Scherrick A, Yamashita K (2000). Lung nodule enhancement at CT: multicenter study. Radiology.

[4] Henschke CI, Yankelevitz DF, Naidich DP, McCauley ID, McGuinness G, Libby MD (2004). CT screening for lung cancer: suspiciousness of nodules according to size on baseline scans. Radiology.

[5] Jeong YJ, Lee KS, Jeong SY, Chung MJ, Shim SS, Kim H (2005). Solitary pulmonary nodule: characterization with combined wash-in and washout features at dynamic multidetector row CT. Radiology.

[6] Gould MK, MacLean CC, Kuschner WG, Rydzak CE, Owens DK (2001). Accuracy of positron emission tomography for diagnosis of pulmonary nodules and mass lesions. JAMA.

[7] Yi CA, Lee KS, Kim B, Choi JY, Kwon JO, Kim H (2006). Tissue characterization of solitary pulmonary nodule: comparative study between helical dynamic CT and integrated PET/CT. J Nucl Med.

[8] Cheran SK, Nielsen ND, Patz EF (2004). False-negative findings for primary lung tumors on FDG positron emission tomography: staging and prognostic implications. AJR.

[9] Nomori H, Watanabe K, Ohtsuka T, Naruke T, Suemasu K, Uno K (2004). Evaluation of F-18 fluorodeoxyglucose (FDG) PET scanning for pulmonary nodules less than 3cm in diameter, with special reference to CT images. Lung Cancer.

[10] Tanaka R, Horikoshi H, Nakazato Y, Seki E, Minato K, Iijima M (2009). Magnetic resonance imaging in peripheral lung adenocarcinoma; correlation with histopathologic features. J Thorac Imaging.

[11] Ohno Y, Sugimura K, Hatabu H (2002). MR imaging of lung cancer. EJR.

[12] Podobnik J, Kocijancic I, Kovac V, Sersa I (2010). 3T MRI in evaluation of asbestos-related thoracic diseases – preliminary results. Radiol Oncol.

[13] Schaefer JF, Vollmar J, Schick F, Vonthein R, Seemann MD, Aebert H (2004). Solitary pulmonary nodules: dynamic contrast-enhanced MR imaging-perfusion differences in malignant and benign lesions. Radiology.

[14] Ohno Y, Hatabu H, Takenaka D, Adachi S, Kono M, Sugimura K (2002). Solitary pulmonary nodules: Potential role of dynamic MR imaging in management: initial experience. Radiology.

[15] Abdel Razek A, Elmorsy A, Elshafey M, Elhadedy T, Hamza O (2009). Assessment of mediastinal tumors with diffusion-weighted single-shot echo-planar MRI. J Magn Reson Imaging.

[16] Ichikawa T, Erturk SM, Motosugi U, Sou H, Iino H, Araki T (2007). High-b value diffusion-weighted MRI for detecting pancreatic adenocarcinoma: Preliminary results. AJR.

[17] Inan N, Kilinc F, Sarisoy T, Gumustas S, Akansel G, Demirci A (2010). Diffusion weighted MR imaging in the differential diagnosis of haemangiomas and metastases of the liver. Radiol Oncol.

[18] Murtz P, Flacke S, Traber F, van den Brink JS, Gieseke J, Schild HH (2002). Abdomen: diffusion weighted MR imaging with pulse-triggered singleshot sequences. Radiology.

[19] Ozsunar Y, Sorensen AG (2000). Diffusion- and perfusion- weighted magnetic resonance imaging in human acute ischemic stroke: technical considerations. Top Magn Reson Imaging.

[20] Matoba M, Tonami H, Kondou T, Yokota H, Higashi K, Toga H (2007). Lung carcinoma: diffusion weighted MR imaging—Preliminary evaluation with apparent diffusion coefficient. Radiology.

[21] Uto T, Takehara Y, Nakamura Y, Naito T, Hashimoto D, Inui N (2009). Higher sensitivity and specificity for diffusion- weighted imaging of malignant lung lesions without apparent diffusion coefficient quantification. Radiology.

[22] Satoh S, Kitazume Y, Ohdama S, Kimula Y, Taura S, Endo Y (2008). Can malignant and benign pulmonary nodules be differentiated with diffusion-weighted MRI?. AJR.

[23] Mori T, Nomori H, Ikeda K, Kawanaka K, Shiraishi S, Katahira K (2008). Diffusion-weighted magnetic resonance imaging for diagnosing malignant pulmonary nodules/masses. Comparison with positron emission tomography. J Thorac Oncol.

[24] Liu H, Liu Y, Yu T, Ye N (2010). Usefulness of diffusion-weighted MR imaging in the evaluation of pulmonary lesions. Eur Radiol.

[25] Qi LP, Zhang XP, Tang T, Li J, Sun YS, Zhu GY (2009). Using diffusion-weighted MR imaging for tumor detection in the collapsed lung: a preliminary study. Eur Radiol.

[26] Abdel Razek A, Fathy A, Abdel Gawad T (2011). Correlation of apparent diffusion coefficient value with prognostic parameters of lung cancer. JCAT.

[27] Koyama H, Ohno Y, Aoyama N, Onishi Y, Matsumoto K, Nagomi M (2010). Comparison of STIR turbo SE imaging and diffusion-weighted imaging of the lung: capability for detection and subtype classification of pulmonary adenocarcinomas. Eur Radiol.

[28] Cotran RS, Kumar V, Robbins SL (1994). Robbins Pathologic Basis of Disease.

[29] Clayton F (1986). Bronchioloalveolar carcinoma: Cell types, patterns of growth, and prognostic correlates. Cancer.

[30] Kanauchi N, Oizumi H, Honma T, Kato H, Endo M, Suzuki J (2009). Role of diffusion-weighted magnetic resonance imaging for predicting of tumor invasiveness for clinical stage IA non-small cell lung cancer. Eur J Cardiothorac Surg.

[31] Henzler T, Schmid-Bindert G, Schoenberg SO, Fink C (2010). Diffusion and perfusion MRI of the lung and mediastinum. Eur J Radiol.

